# Deciphering RNA-Recognition Patterns of Intrinsically Disordered Proteins

**DOI:** 10.3390/ijms19061595

**Published:** 2018-05-29

**Authors:** Ambuj Srivastava, Shandar Ahmad, M. Michael Gromiha

**Affiliations:** 1 Department of Biotechnology, Bhupat and Jyoti Mehta School of Biosciences, Indian Institute of Technology Madras, Chennai 600 036, Tamilnadu, India; ambuj.88.in@gmail.com; 2 School of Computational and Integrative Sciences, Jawaharlal Nehru University, New Delhi 110 067, India; shandar@jnu.ac.in

**Keywords:** intrinsically disorder proteins, disorder-to-order regions, protein–RNA interactions, unstructured proteins

## Abstract

Intrinsically disordered regions (IDRs) and protein (IDPs) are highly flexible owing to their lack of well-defined structures. A subset of such proteins interacts with various substrates; including RNA; frequently adopting regular structures in the final complex. In this work; we have analysed a dataset of protein–RNA complexes undergoing disorder-to-order transition (DOT) upon binding. We found that DOT regions are generally small in size (less than 3 residues) for RNA binding proteins. Like structured proteins; positively charged residues are found to interact with RNA molecules; indicating the dominance of electrostatic and cation-π interactions. However, a comparison of binding frequency shows that interface hydrophobic and aromatic residues have more interactions in only DOT regions than in a protein. Further; DOT regions have significantly higher exposure to water than their structured counterparts. Interactions of DOT regions with RNA increase the sheet formation with minor changes in helix forming residues. We have computed the interaction energy for amino acids–nucleotide pairs; which showed the preference of His–G; Asn–U and Ser–U at for the interface of DOT regions. This study provides insights to understand protein–RNA interactions and the results could also be used for developing a tool for identifying DOT regions in RNA binding proteins.

## 1. Introduction

Intrinsically disordered proteins lack stable three-dimensional structures under physiological conditions and are known to perform important roles in several processes including signalling, enzymatic activity, and gene regulation [1,2]. To perform these functions, disordered regions interact with protein, RNA, DNA, and other small molecules to gain ordered structures [3,4]. Experimentally, interactions mediated by IDRs can be observed using NMR and X-ray crystallography. However, because of poor resolution, problems in crystallization, and high time and resource consumption, computational methods are necessary to identify disorder-mediated interactions [5,6].

Several methods have been developed for understanding the disorderness of proteins using sequence or structural information [7,8,9,10]. In addition, the transition of disorder-to-order regions in protein–protein interactions (PPI) is well studied experimentally and computationally [8,9,10,11,12]. For example, LMO4, a putative breast oncoprotein, interacts with various tandem LIM-domain containing proteins mediated by disordered regions [13]. BRCA1, a tumour suppressor protein, helps in binding with multiple protein and DNA partners by its central disorder region of ~1500 amino acids [14]. Recently, Papadakos et al. [15] showed that inducing intrinsic disorder in high-affinity protein–protein interactions reduces the affinity of binding.

Many proteins contain disordered regions and some of the regions attained ordered structures after binding to their cognate substrates, which are also known as MoRF (Molecular Recognition Features) segments [16,17]. Sugase et al. [18] have shown that folding and binding of IDPs or IDRs are coupled processes. Furthermore, binding partners are also shown to influence affinity and kinetics of binding. The flexibility of IDPs helps them to bind with multiple partners and have co-operative interactions [19]. Although induced fit and conformational selection processes are proposed explanations for the coupling of folding and binding, the exact model which is preferred by IDPs is not known [11,20].

The dynamics of the RNA molecule makes it more amenable to interact with disorder-mediated protein–RNA interactions [21]. The recognition of the protein–RNA complex has been experimentally studied using EMSA, yeast-3-hybrid assay, pull-down assay and CLIP [22,23]. On the other hand, plenty of tools have been developed to identify binding sites in RNA-binding proteins [24,25,26,27,28,29,30,31,32]. All these methods use the information in their sequence to compute the feature and/or evaluate the performance. Recently, Peng and Kurgan [33] developed a webserver for prediction of disorder-mediated interactions in RNA, DNA and protein–protein complexes. However, the knowledge for understanding the mechanisms or factors responsible for binding of disordered region with RNA has not yet been completely explored.

In this work, we constructed a dataset for protein–RNA complexes (provided in supplementary information), which are involved in disorder-to-order transitions. Utilizing the dataset, we analyzed the number and size of DOT regions in protein–RNA complexes, preference of residues involved in binding in DOT regions, secondary structure, solvent accessibility, pair preference at the interface, preference in different secondary structures of RNA, and interaction energy between protein and RNA DOT and non-DOT regions at the interface.

## 2. Results and Discussion

Our dataset contains a total of 23,452 and 2412 residues in non-ribosomal and ribosomal protein–RNA complexes. Among them, 1175 (5%) and 155 (6.4%) residues are found to be in DOT regions in non-ribosomal and ribosomal complexes, respectively. The residues binding with RNA are obtained by using 3.5 and 6 Å distance cut-offs and similar trends are obtained. Therefore, we have presented the results with 3.5 Å and those for 6 Å are shown in supplementary material. 

### 2.1. Number of DOT Regions in Protein–RNA Complexes and Length of DOT Regions

**Figure 1:**
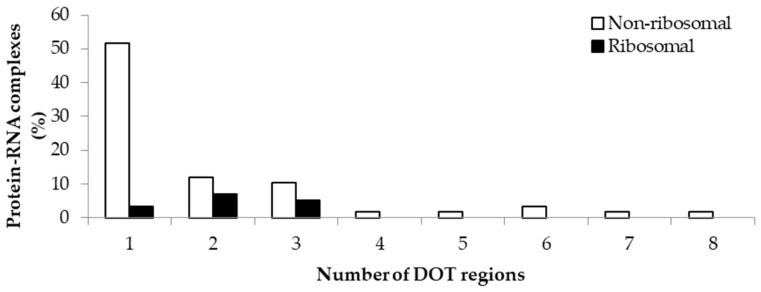
Percentage of protein–RNA complexes containing different number of DOT regions.

The variation in the number of DOT regions in non-ribosomal and ribosomal complexes is shown in Figure 1. We observed that most of the complexes have less than three DOT regions (88% in non-ribosomal and 100% in ribosomal complexes). Most non-ribosomal proteins have one DOT region, whereas ribosomal proteins have mostly two or more DOT regions. In addition, at most eight DOT regions per complex are found in our dataset.

**Figure 2:**
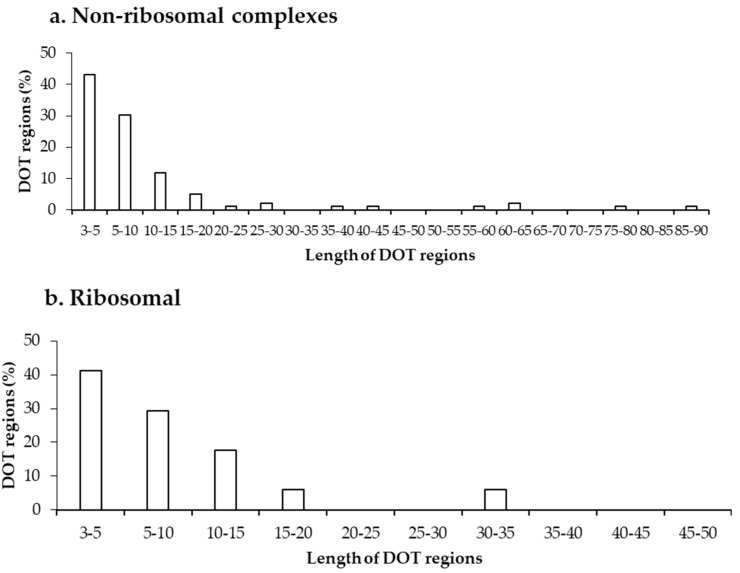
Length distribution of DOT regions in protein–RNA complexes in (**a**) non-ribosomal and (**b**) ribosomal complexes.

Further, we analysed the length of each DOT region in non-ribosomal and ribosomal protein–RNA complexes, which shows that most DOT regions are short, as shown in Figure 2. In both non-ribosomal and ribosomal complexes, more than 70% of DOT regions have three to 10 residues and very few (only 5) regions have a length of more than 50 residues. This leads to a speculation that only a small conformational change might be required for bringing shape complementarity in protein–RNA complexes and these small DOT regions help in obtaining the same. 

### 2.2. Binding Frequency of Residues at DOT Regions

**Figure 3:**
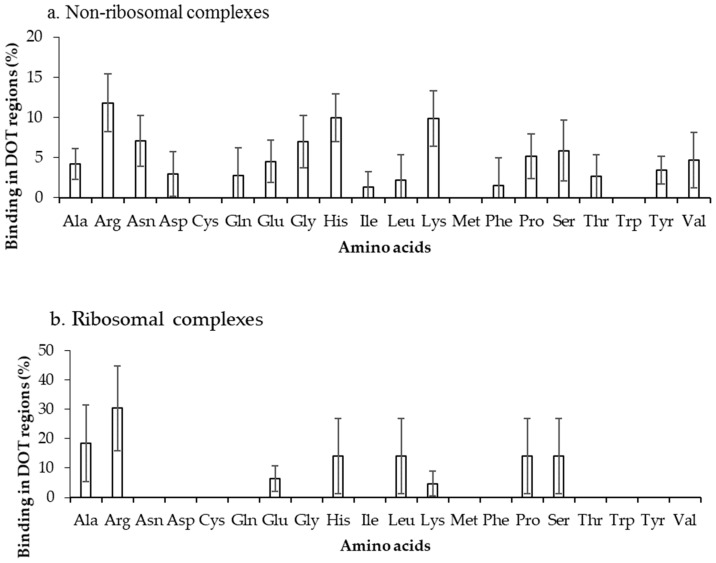
Amino acid frequency of binding in the DOT region for (**a**) non-ribosomal and (**b**) ribosomal protein–RNA complexes.

The binding frequencies of residues in DOT regions using 3.5 Å (NR3.5 and RB3.5) and 6 Å (NR6 and RB6) distance cut-offs are shown in Figure 3 and Figure S2, respectively. We observed that among all positively charged residues (Arg, Lys and His), Arg and Lys have high preference for binding in both NR3.5 (Figure 3a) and NR6 (Figure S2a) datasets. Interestingly, only eight and 13 among 20 residues are observed in binding DOT regions at RB3.5 (Figure 3b) and RB6 (Figure S2b) datasets, and Arg has the highest frequency of binding. Cys, Met, and Trp in DOT regions are not involved in binding with RNA, whereas in ordered complexes 0.97%, 4.52%, and 5.54% of Cys, Met, and Trp are involved in binding, respectively. The comparison of binding site residues in DOT regions and the whole protein showed an expected presence of 1.5% and 2.7% of Met and Trp, respectively, in the interface of the DOT region. These results showed that the non-occurrence of Met and Trp at the interface of the DOT regions is statistically significant.

**Figure 4:**
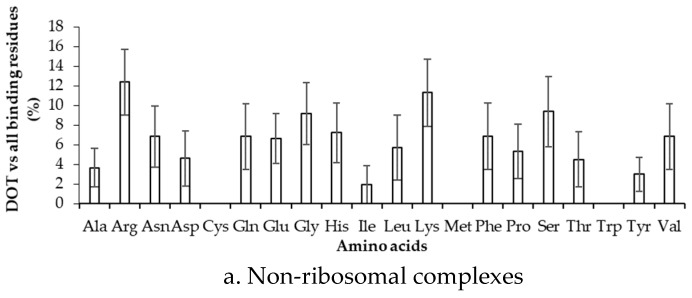

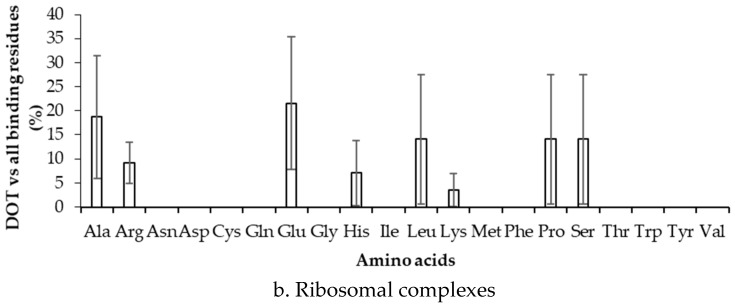
Frequency of DOT regions by contact residues for (**a**) non-ribosomal and (**b**) ribosomal complexes.

We have computed the preference of binding of residues in DOT regions by dividing the number of residues in DOT regions with the total number of binding residues, and the results are presented in Figure 4 and Figure S3 for 3.5 Å (NR3.5 and RB3.5 datasets) and 6 Å (NR6 and RB6 datasets), respectively. In Figure 4a, high frequency of Arg, Gly, Lys, and Ser (*z*-score > 1) is observed for the NR3.5 dataset, which suggests that these residues are more probable to contact DOT regions with respect to all residues in contact with RNA. However, for the NR6 dataset (Figure S3a), the result is only consistent for Lys, and two other residues (Glu and Pro) show high binding frequency. In ribosomal protein complexes with 3.5 Å and 6 Å, Ala & Glu, and Glu & Tyr have high frequencies, respectively (Figure 4b and Figure S3b).

### 2.3. Binding Propensity of Residues at DOT Region

**Figure 5:**
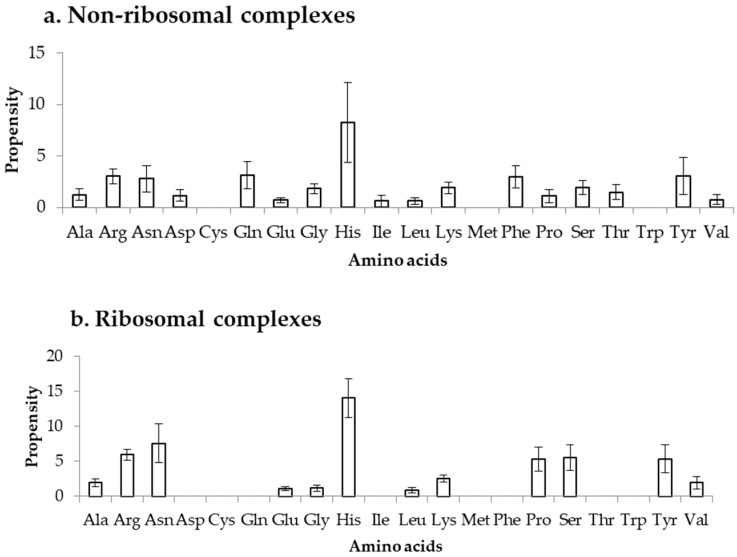
Propensity for amino acids in (**a**) non-ribosomal and (**b**) ribosomal complexes.

Propensity is calculated by normalizing the binding frequency of residues in DOT regions with the overall frequency of the respective residues to be in a protein, using Equation (3). This can measure the bias in binding of residues in DOT regions, independent of their count in DOT regions. We have calculated the propensity of amino acids to be in DOT regions using distance cut-offs of 3.5 Å and 6 Å and the results are shown in Figure 5 and Figure S4, respectively. In the NR3.5 (Figure 5a) dataset, His, Arg, Asn, Gln, Phe, and Tyr have high propensity of binding, whereas in ribosomal proteins (RB3.5 dataset; Figure 5b), only His showed a high propensity. In the NR6 (Figure S4a), His has high propensity, whereas Asn, His and Tyr have high propensity in the RB6 (Figure S4b) dataset. Similarly, high propensity for binding is observed for positively charged residues along with Tyr and Phe in protein–RNA complexes [34]. On the other hand, among all charged residues only Arg has high tendency to bind with DOT regions in protein–protein complexes [35]. Furthermore, non-specific interactions occurred frequently in protein–protein complexes, which is not a common trend in the binding residues of DOT regions in protein–RNA complexes. Therefore, we can infer that the preferred residues at DOT regions are specific in protein–RNA complexes and, especially, charged interactions are important in DOT regions for binding with RNA. 

### 2.4. Comparison of Frequency of Binding in the DOT Region and Other Residues of a Protein

**Figure 6:**
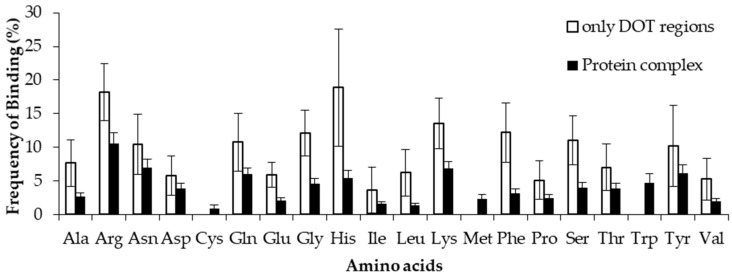
Binding frequency of each amino acid in the DOT region and in the overall protein for non-ribosomal complexes using the 3.5 Å cut-off.

To estimate the difference between binding in DOT regions and other part of proteins, we calculated the binding frequency of amino acids in these regions, as shown in Figure 6 and Figure S5. Amino acids significantly differ in their binding with RNA in DOT regions and in the complete protein (*p*-value for the mean is less than 0.01). In non-ribosomal proteins, when the 3.5 Å cut-off is considered, nonpolar and aromatic residues mostly have high frequency values in the DOT regions than in the overall protein. All the frequencies are observed to be significant when statistical analysis is performed for the bootstrapped sample of the frequencies (*p*-value is less than 0.01). Residues such as His, Phe, and Leu are found to have a more than 3-fold increase in the frequency of binding in the DOT regions than in other parts of the proteins. A similar trend is observed in the NR6 dataset (Figure S5).

### 2.5. Amino Acid Contact Frequency with Nucleotides

**Figure 7:**
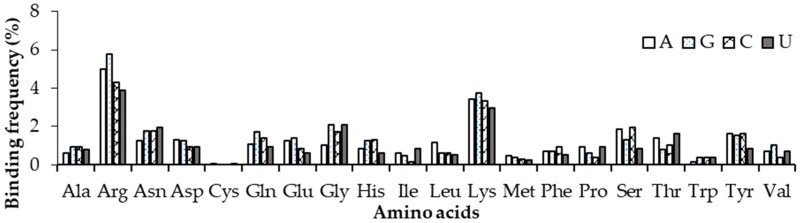
Normalized amino acid nucleotide contact frequency in non-ribosomal protein–RNA complexes at 3.5 Å.

We have also analysed amino acid contacts with each nucleotide in non-ribosomal complexes using 3.5 Å and 6 Å distance cut-offs for contacting residues and the results are shown in Figure 7 and Figure S6. In the 3.5 Å distance criterion, Arg and Lys have a high frequency to bind with nucleotides. Arg and Lys are observed to have the most and least binding frequencies with Guanine and Uracil, respectively. Whereas in the 6 Å criterion, almost the same frequency of binding is observed for Arg and Lys with Adenine, Guanine and Cytosine nucleotides; least binding was observed in the Uracil nucleotide. When compared with the results presented for ordered protein–DNA and protein–RNA complexes in our earlier works, Arg, Lys, Trp, and Tyr were favoured by RNA and Arg was selected by DNA-binding proteins together with Guanine in DNA and Uracil in RNA–protein complexes [36].

### 2.6. Secondary Structure of DOT and RNA-Interacting DOT Residues

The secondary structures of DOT residues are quantified to study the bias of residues to have a specific secondary structure in binding and non-binding regions and data are presented in Table 1 and Table S1 for NR3.5 and NR6 datasets, respectively. In the NR3.5 dataset, all the DOT residues have lower and higher preference in sheet (15%) and other structure class (8%), respectively. Interestingly, in DOT residues, binding with RNA molecules, strand-forming residues have a higher preference (15.2%) as compared to helical (8.6%) and other regions (8.9%).

### 2.7. Relative Solvent Accessibility of DOT Residues 

The spatial arrangement of DOT residues is further explored by solvent accessibility calculation and the result is shown in Table 2. Comparison of RASA of DOT regions and complete protein–RNA complex revealed that in DOT regions, solvent accessibility of every amino acid is more than that of other amino acids of a protein. As expected, charged residues have low fold difference (1.18 to 1.28) in RASA in DOT regions and the complete protein. However, most hydrophobic residues (Ala, Cys, Ile, Leu, Met, Phe, Tyr, and Val) have about 1.8 to 2 folds higher RASA in DOT regions than the complete protein, Met has the highest difference. On the other hand, the mean solvent accessibility of DOT regions of proteins is 44 Å^2^, which is similar to the average RASA of binding DOT regions (43 Å^2^) of protein–protein complexes [17].

### 2.8. Number of Residues in Contact with Nucleotides in the DOT Region and in Entire Protein

Among 1175 residues in DOT regions in our dataset, only 96 (8.17%) and 268 (22.81%) are in contact with nucleotides in the NR3.5 and the NR6 dataset, respectively. Almost all the residues have a similar tendency of binding with nucleotides in proteins, ranging between 20% to 29%, as shown in Table 3 and Table S2. However, the number of nucleotides interacting with DOT residues is somewhat different, that is, the range of interaction is 18 to 33%. The DOT residues are more likely to bind with Guanine (20.4%), followed by Cytosine and Uracil, than to binding with Adenine (13.1%).

### 2.9. Secondary Structure of Nucleotides Interacting with DOT Residues

Further, we have classified the nucleotides based on location and contacts with DOT residues and preference of amino acids in a protein and the results are presented in Table 4 and Table S3. Among all secondary structures formed by nucleotides, unpaired bases are most likely to bind with DOT residues. Specifically, we observed that A and U in unpaired regions prefer to interact with DOT residues, whereas C and G in unpaired and base-paired positions interact with DOT residues with a similar preference. G and C also interact with DOT residues in pseudoknot secondary structure, whereas A and U are least likely to exist in pseudoknot form when bound to DOT regions. 

### 2.10. Interaction Energy of DOT Residues with Nucleotides

We have computed the interaction energy between amino acids and nucleotides in DOT and ordered regions at the binding interface and the results are presented in Table 5. Most of the amino acids have stronger interactions with nucleotides in ordered regions than DOT regions. However, we noticed that some combinations of amino acid–nucleotide pairs have favourable energy when interacting with DOT regions. For example, Arg, His, Ile, Leu, Val, and Phe interact with G, His, Ser, and Val with C, and Asn, Asp, Gly, Ile, Leu, and Ser with U. In addition, hydrophobic residues Ile, Leu, and Val have more favourable interactions with G at DOT regions than others. Since Arg and Lys are important for protein–RNA complex formation through electrostatic interactions these residues have stronger energies in ordered regions than DOT regions. On the other hand, His in the DOT region has favourable energy with G and C. These differences in energy could be important to understand the interactions between DOT regions and the RNA molecule, which might also be used to distinguish the RNA binding residues of proteins in DOT and other regions.

**Figure 8:**
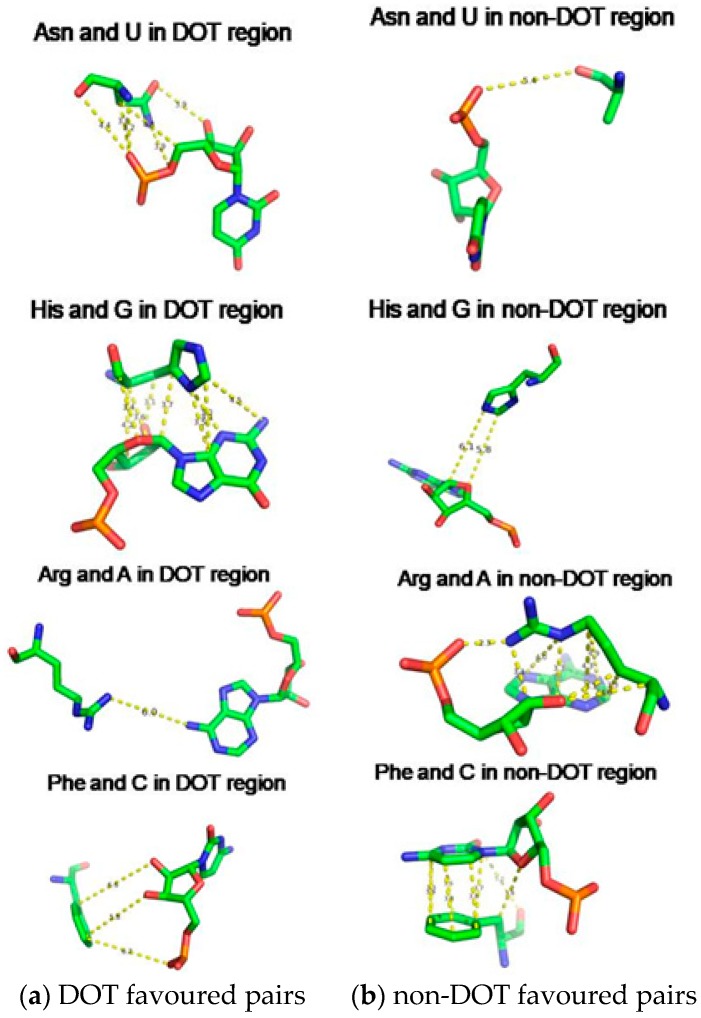
Amino acid showing (**a**) strong interaction in DOT and weak interaction in non-DOT regions and (**b**)weak interaction in DOT and strong interaction in non-DOT regions.

We have compared the interaction energy of amino acid–nucleotide pairs in the interface of DOT and other regions and two typical examples are shown in Figure 8. We noticed a wide range of interactions such as stacking, cation-π, electrostatic, and van der Waals interactions at the interface. Most favourable energy is observed for Asn and His with U (−3.26 kcal/mol) and G (−5.44 kcal/mol), respectively, in DOT regions (Figure 8a). On the other hand, Arg and Phe have favourable energy with A (−8.49 kcal/mol) and C (−4.88 kcal/mol), respectively, in non-DOT regions.

## 3. Materials and Methods

**Figure 9:**
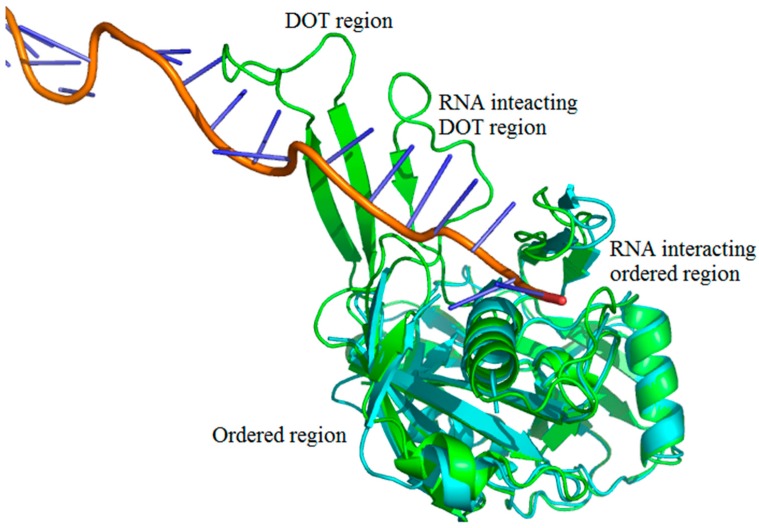
Representation of disorder-to-order mediated interactions. Free protein, RNA, and complex (CRISPR-Cas RNA Silencing Cmr Complex) are shown in cyan, orange and green, respectively. The PDB IDs are 4H4K:A (free protein), 3XIL:I (RNA of protein–RNA complex) and 3XIL:B (RNA-bound protein). The disorder-to-order transition (DOT) region can be clearly seen in green with a missing overlapping region of free protein.

We adopted the following protocol to obtain a set of protein–RNA complexes with disorder-to-order transition (DOT) regions: (i) Downloaded the protein–RNA complexes from PDB and NDB databases (www.rcsb.org) [37,38,39]; (ii) Clustered all the protein–RNA complexes with 30% sequence identity cut-off using CD-Hit suite [40]; (iii) Performed BLAST search (using 99% identity cut-off) of protein sequences to obtain free proteins corresponding to each protein–RNA complex [41,42]. The free proteins have the same sequences as the protein part of protein–RNA complexes but crystallized without RNA. Note that free proteins contain unique PDB IDs, which is distinct from the protein–RNA complex; (iv) Disordered residues are obtained from missing residues information in the protein–RNA complex and free protein pairs by locating “REMARK 465” statement in the protein structure file; (v) DOT residues are isolated by comparing the disorder residues of free and protein–RNA complex pairs such that the residue is ordered in the protein–RNA complex but disordered in free protein. Note that only the regions having 3 or more continuous DOT residues are considered. The final dataset contains 101 DOT regions in 52 proteins and complete data are given in supplementary information. The representation of DOT and ordered region in a typical protein–RNA complex (PDB ID: 4H4K) is shown in Figure 9.

### 3.1. Number of DOT Regions and Their Lengths

The number of DOT regions and their lengths are obtained by counting the number of non-consecutive and consecutive residues, respectively, using custom build python scripts. 

### 3.2. DOT Residues in Contact with RNA

The residues in contact with RNA molecules are obtained by using distance cut-offs mentioned in literature, that is, 3.5 Å and 6 Å [43,44,45]. Binding residues in DOT regions are obtained by taking common residues in the DOT dataset and RNA contacting residues. We have classified protein–RNA complexes in non-ribosomal and ribosomal classes because of the difference in their interaction pattern, number of interacting amino acids, and residue bias in them [46]. Therefore, using the type of complex and distance cut-off for interacting residues, we divided protein–RNA complexes into four different datasets: (1) NR3.5: non-ribosomal complex with a contact distance of 3.5 Å; (2) RB3.5: ribosomal complex with a contact distance of 3.5 Å; (3) NR6: non-ribosomal complex with a contact distance of 6 Å; and (4) RB6: ribosomal complex with a contact distance of 6 Å.

We computed the frequency of each DOT residue involved in binding using the Equation (1).

(1) $$\mathrm{Frequency}\;\mathrm{of}\;\mathrm{binding}\;\mathrm{residues}\;\mathrm{in}\;\mathrm{DOT}\;\mathrm{region}=\frac{N_{ib}}{N_{id}}$$

where *N_ib_*: number of *i*th residues binding in the DOT region and *N_id_*: number of *i*th residues in DOT.

Moreover, the differences in the frequency of binding residues in DOT regions and in the protein complexes are obtained.

### 3.3. Frequency of Binding in DOT and Other Residues 

We also computed the frequency of residues binding in DOT regions over all the binding residues by using Equation (2), an error bar is plotted using the bootstrap method by randomly re-sampling an equal sized data with a replacement 1000 times.

(2) $$\mathrm{Frequency}\;\mathrm{of}\;\mathrm{binding}\;\mathrm{by}\;\mathrm{contact}\;\mathrm{residues}=\frac{N_{ibd}}{N_{ib}}$$

where *N_ibd_*: number of *i*th residues binding in DOT region; *N_ib_* is number of *i*th residues binding with RNA in complete protein.

### 3.4. Propensity of Binding Residues in DOT Region

The normalization of frequency of residues present in DOT regions by individual residue frequency provides the tendency of a residue in DOT regions. Accordingly, propensity values are calculated using the following equation:

(3) $$\mathrm{Propensity}(I)=\frac{N_{ibd}/N_{id}}{N_{ip}/N_{p}}$$

where Propensity (I): propensity of *i*th residue; *N_ibd_*: number of *i*th residue binding in DOT region; *N_id_*: number of *i*th residue in DOT regions; *N_ip_*: number of *i*th residue in protein; *N_p_*: number of residues in protein.

### 3.5. Boot Strap Sampling

To obtain the standard error in frequency and propensity calculations, bootstrap sampling is performed. In this technique all the protein–RNA complexes are sampled randomly and each sample contains complexes equal to the number of protein–RNA complexes. Therefore, each sample will have redundancy of some complexes and will be devoid of some complexes. In this manner, we have created 1000 samples on which the calculations are performed.

### 3.6. Relative Average Solvent Accessibility (RASA)

The DOT residues buriedness is analysed by the NACCESS [47] program and the RASA of each residue is calculated by using Equation (4).

(4) $$\mathrm{RASA}=\frac{A_{ibd}}{\sum_{i=1}^{n}\left(A_{ibd}\right)}$$

where *A_ibd_*: RASA of *i*th residue binding with RNA in DOT region; *n*: number of DOT residues in a protein–RNA complex.

### 3.7. Secondary Structure of Protein and RNA 

Secondary structure of both proteins and RNA molecules are analysed by DSSP and DSSR programs, respectively [48,49]. The DSSR program gives dot bracket notation of secondary structure of RNA as shown in Figure S1, in which “.” represents unpaired nucleotide, “(” or “)” represent paired bases, and “{” or “}” or “[” or “]” or “<” or “>” represent pseudoknot bases.

### 3.8. Binding Preference of Nucleotides for Amino Acids

The binding preference of nucleotide with DOT residues has been calculated by counting the occurrence of nucleotides–amino acid interacting pairs under the distance of 3.5 Å.

### 3.9. Interaction Energy between Amino Acids and Nucleotides at Binding Interface

The interaction energy of amino acids with nucleotides is computed using van der Waals and coulombs potential using AMBER force field [50]. It is given by

(5) $$\mathrm{Energy}=\sum \left[\left(\frac{A_{ij}}{r_{ij}{}^{12}}-\frac{B_{ij}}{r_{ij}{}^{6}}\right)+\frac{q_{i}q_{j}}{\varepsilon r_{ij}}\right]\;$$

where, *A_ij_* = ε_ij_* (R_ij_*)^12^ and *B_ij_* = 2 ε_ij_* (R_ij_*)^6^; R_ij_* = (R_i_* + R_j_*); and ε_ij_* = (ε_i_* ε_j_*)^1/2^; R* and ε* van der Waals radius and well depth, respectively, and these parameters are obtained from Gromiha et al. [51]; *q_i_* and *q_j_* is the charge on atom *i* and *j*, respectively and *R_ij_* is the distance separating atom *i* and *j*.

## 4. Conclusions

The analysis of DOT regions in protein–RNA complexes revealed that in each complex these regions are generally small in size. Electrostatic interactions are found to be important, with the involvement of positively charged residues (Arg, Lys and His) in DOT regions. Among nucleotide–amino acid pairs, guanine–Arg and uracil–Lys pairs are identified to be the most and the least preferred ones at the interface, respectively. Generally, nucleotides prefer to bind DOT regions than other regions of protein. Further, DOT regions are significantly more exposed to solvent than other residues of protein–RNA complexes. Specifically, hydrophobic residues have higher difference in RASA of DOT regions and complete proteins. DOT regions are preferred to form coils, turns, and bends than regular secondary structures such as helices and strands. On the RNA side, DOT residues prefer to bind unpaired A and U and paired regions of C and G. In pseudoknot condition, mostly C and G interact with DOT residues. The interaction energy calculations revealed the types of interactions and preferred amino acid-nucleotide pairs at the interface based on energy.

The frequencies and propensities obtained in the present study could be used for discriminating DOT binding residues from other residues. Further, the location of DOT binding residues based on solvent accessibility and secondary structure of protein and RNA along with energy calculations may help to understand the recognition mechanism. 

We obtained the DOT regions by comparing 3D coordinates of the missing residues in protein–RNA complexes and their respective free proteins. This might be an under representation of DOT regions since the structures solved by crystallization often stabilize the residues and reduce the native disorder. Hence, the disordered residues having 3D coordinates in free proteins are not considered. The current study can further be refined with the availability of more numbers of protein–RNA complexes and the improvements in structure determination techniques. In addition, development of disorder specific databases for protein–nucleic acid complexes with large datasets could enhance the confidence level of the result reported in the present study.

## Figures and Tables

**Table 1:** Secondary structure of all DOT residues and residues binding with RNA in DOT regions in the NR3.5 dataset.

Secondary Structure	Number of Binding Residues in DOT Regions (*N_idt_*)	Number of Residues in DOT Region (*N_d_*)	Relative Binding in DOT Regions (%)
Helix	25 (22.12)	288 (24.51)	8.6
Sheet	22 (19.47)	145 (12.34)	15.2
Others (coil, turn, bend)	66 (58.41)	742 (63.15)	8.9

Percentage is mentioned in the parenthesis. Relative binding in DOT regions are calculated by *N_idt_*/*N_d_* × 100.

**Table 2:** Relative average solvent accessibility (RASA) of DOT residues and all residues in non-ribosomal protein–RNA complexes.

Amino Acids	RASA in DOT Regions	RASA in Complete Protein	Fold Difference
Ala	44.743	23.305	1.920
Arg	52.168	40.822	1.278
Asn	63.583	42.721	1.488
Asp	56.552	43.811	1.291
Cys	22.32	11.426	1.953
Gln	47.805	38.988	1.226
Glu	53.688	47.838	1.122
Gly	51.599	35.272	1.463
His	43.229	35.372	1.222
Ile	26.618	14.692	1.812
Leu	32.007	16.374	1.955
Lys	58.529	49.520	1.182
Met	41.13	20.391	2.017
Phe	32.481	17.519	1.854
Pro	56.463	38.230	1.477
Ser	54.574	34.622	1.576
Thr	49.852	31.074	1.604
Trp	21.571	19.029	1.134
Tyr	44.579	24.752	1.801
Val	31.433	17.405	1.806

**Table 3:** Number of interaction of nucleotides with DOT residues and with complete protein at 3.5 Å.

Nucleotides	Number of Nucleotide in Contact with DOT Regions (*N_idt_*)	Number of Nucleotides in Contact with Any Residue of Proteins (*N_prot_*)	Relative Contact in DOT Regions (%)
A	18 (18.75)	137 (25.66)	13.1
C	26 (27.08)	131 (24.53)	19.8
G	32 (33.33)	157 (29.40)	20.4
U	20 (20.83)	109 (20.41)	18.3

Percentage is mentioned in the parenthesis. Relative contact in DOT regions are calculated by *N_idt_*/*N_prot_* × 100.

**Table 4:** Preference of nucleotides in different secondary structures to bind with DOT residues.

Nucleotides	Secondary Structure	Number of Nucleotide in Contact with DOT Regions (*N_idt_*)	Number of Nucleotides in Contact with Any Residue of Proteins (*N_prot_*)	Relative Contact in DOT Regions (%)
A	Unpaired	12 (12.50)	106 (19.56)	11.01
A	Basepaired	6 (6.25)	30 (5.54)	20.00
A	Pseudoknot	0 (0)	0 (0)	0
C	Unpaired	8 (8.33)	70 (12.92)	11.42
C	Basepaired	17 (17.71)	59 (10.89)	28.81
C	Pseudoknot	1 (1.04)	5 (0.92)	20.00
G	Unpaired	16 (16.67)	87 (16.05)	18.39
G	Basepaired	15 (15.63)	71 (13.10)	21.13
G	Pseudoknot	1 (1.04)	4 (0.74)	25.00
U	Unpaired	15 (15.63)	81 (14.94)	18.51
U	Basepaired	5 (5.21)	29 (5.35)	17.24
U	Pseudoknot	0 (0)	0 (0)	0
All	Unpaired	51 (53.13)	344 (63.47)	14.83
All	Basepaired	43 (44.79)	189 (34.87)	22.75
All	Pseudoknot	2 (2.08)	9 (1.66)	22.22

Percentage is mentioned in parenthesis. Relative contacts in DOT regions are calculated by *N_idt_*/*N_prot_* × 100.

**Table 5:** Interaction energy between amino acids and nucleotides in DOT regions.

Amino Acids	A	G	C	U
Ala	−0.62 (−0.55)	−0.34 (−0.57)	−0.49 (−0.53)	−0.55 (−0.64)
Arg	−0.36 (−1.23)	**−1.15** (−0.83)	−0.89 (−0.95)	**−1.06** (−0.98)
Asn	−0.45 (−0.68)	−0.59 (−0.73)	−0.48 (−0.83)	**−1.85** (−0.82)
Asp	−0.75 (−0.74)	−0.39 (−0.79)	−0.19 (−0.56)	**−1.40** (−0.92)
Cys	0.00 (−0.87)	−0.01 (−0.03)	−0.03 (−1.10)	−0.63 (−1.13)
Gln	−0.15 (−0.87)	−0.57 (−0.74)	−0.08 (−0.84)	−0.36 (−0.71)
Glu	−0.72 (−0.80)	−0.41 (−0.64)	−0.43 (−0.62)	−0.68 (−0.59)
Gly	−0.28 (−0.47)	−0.37 (−0.69)	−0.58 (−0.57)	**−1.07** (−0.79)
His	−0.81 (−1.17)	**−2.13** (−1.41)	**−1.53** (−1.21)	−0.70 (−1.01)
Ile	−0.60 (−0.64)	**−1.63** (−0.80)	−0.54 (−0.50)	**−1.33** (−0.76)
Leu	−0.35 (−0.75)	**−1.19** (−0.50)	−0.42 (−0.49)	−0.54 (−0.41)
Lys	−0.74 (−0.76)	−0.86 (−0.83)	−0.66 (−0.90)	−0.83 (−0.83)
Met	−0.64 (−1.05)	−0.07 (−0.75)	−0.16 (−1.03)	−0.83 (−1.19)
Phe	−0.81 (−1.03)	**−1.12** (−0.89)	−0.54 (−1.32)	−0.24 (−1.42)
Pro	−0.88 (−0.83)	−0.60 (−0.88)	−0.62 (−0.91)	−0.69 (−1.00)
Ser	−0.79 (−0.77)	−0.29 (−0.56)	**−1.24** (−0.71)	**−1.41** (−0.68)
Thr	−0.39 (−0.68)	−0.66 (−0.56)	−0.67 (−0.64)	−0.53 (−1.00)
Trp	**−1.15** (−1.10)	0.00 (−1.64)	0.00 (−0.99)	0.00 (−1.34)
Tyr	**−1.53** (−1.36)	−1.11 (−1.42)	−0.63 (−1.05)	−0.16 (−1.09)
Val	−0.38 (−0.70)	−0.66 (−0.53)	−0.64 (−0.53)	−0.52 (−0.76)

Interaction energy for non-DOT residues is mentioned in the parenthesis. Amino acid–nucleotide pairs with favourable interaction energies in DOT regions are shown in bold.

## Supplementary Materials

Supplementary materials can be found at http://www.mdpi.com/1422-0067/19/6/1595/s1.
