# Supplementary material for: Deciphering RNA-Recognition Patterns of Intrinsically Disordered Proteins
Source: Int J Mol Sci. 2018 May 29;19(6):1595. doi: 10.3390/ijms19061595 (PMC6032373; doi:10.3390/ijms19061595)
Supplement: Supplementary file 1 [file ijms-19-01595-s001.pdf]

International Journal of Molecular Sciences

MDPI

Supplementary Information

Dataset

```
#Information of DOT regions of protein-RNA complexes

#File format
#Protein-RNA complex --> free protein
#DOT residue name_Position of residue in protein-RNA complex
#Each entry is separated by "----------------"
>2B63:A --> 3CQZ:A
VAL_2 GLY_3 GLN_4 GLN_5 MET_41 ASP_42 GLU_43 THR_44 GLN_45 THR_46
ARG_47 SER_249 ILE_250 SER_251 PHE_252 ASN_253 GLU_254 SER_255 GLN_256
ARG_257 GLY_258 GLU_259 ASP_260 ASP_261 LEU_262 ASP_305 ASN_306
ASP_307 ILE_308 ALA_309 GLY_310 GLN_311 PRO_312 GLN_313 ALA_314
LEU_315 GLN_316 LYS_317 SER_318 GLY_319 ARG_320 PRO_321 VAL_322
LYS_323 SER_324 ILE_325 ARG_326 ALA_327 ARG_328 LEU_329 LYS_330
GLY_331 LYS_332 GLU_333 GLY_334 ARG_335 ILE_336 ARG_337 GLY_338
ASN_339 LEU_340 MET_341 GLY_342 LYS_343 ARG_344 VAL_345 PHE_1389
ASN_1390 ARG_1391 SER_1392 ASN_1393 THR_1394 GLY_1395 ALA_1396
LEU_1397 VAL_1451 LYS_1452 TYR_1453 MET_1454 PRO_1455
----------------
>4GZY:D --> 3EQL:N
PRO_208 ARG_209 ARG_210 VAL_211 ARG_212 VAL_213 GLU_214 TYR_215
VAL_216 THR_340 GLU_341 PRO_342 LYS_343 ASP_344 TYR_345 ARG_346
VAL_347 GLN_348 PRO_349 HIS_350 MET_351 ASN_352 VAL_353 VAL_354
VAL_355 PRO_356 GLU_357 GLY_358 ALA_359 ARG_360 VAL_361 GLU_362
ALA_363 GLY_364 ASP_365 LYS_366 ILE_367 VAL_368 ALA_369 ALA_370
ILE_371 ASP_372 PRO_373 GLU_374 GLU_375 GLU_376 VAL_377 ILE_378
ALA_379 GLU_380 ALA_381 GLU_382 GLY_383 VAL_384 VAL_385 HIS_386
LEU_387 HIS_388 GLU_389 PRO_390
----------------
>4S20:C --> 5EZK:C
TYR_3 SER_4 TYR_5 THR_6 GLU_7
----------------
>3AVU:A --> 3AGQ:A
ALA_2 GLU_3 ILE_4
----------------
>1R9S:B --> 3CQZ:B
SER_248 ARG_249 PHE_250 TYR_431 MET_432 GLN_433 ARG_434 THR_435
VAL_436 GLU_437 ALA_713 GLU_714 ALA_715 TYR_866 GLY_867 MET_868
SER_869 ASN_881 THR_882 LEU_883 ASP_1100 ASP_1101 LYS_1102 ILE_1103
```

```
HIS_1104 ALA_1105 ARG_1106 ALA_1107 ARG_1108 GLY_1109 PRO_1110
MET_1111 GLN_1112 VAL_1113 LEU_1114 THR_1115 ARG_1116 GLN_1117
PRO_1118 VAL_1119 GLU_1120 GLY_1121 ARG_1122 SER_1123 ARG_1124
ASP_1125 GLY_1126 LYS_1174 LEU_1175 ASN_1176 ARG_1222 ASP_1223
PHE_1224
----------------
>3ICQ:T --> 3IBV:B
ALA_794 PRO_795 GLN_796 GLY_797 THR_798 ALA_847 ASN_848 LEU_849
VAL_850 TRP_870 GLY_871 GLY_872 LYS_873 ASP_874 GLY_875 ILE_876
ALA_877 PRO_894 VAL_895 ASN_896 VAL_940 ASN_941 PHE_942 PRO_943
ASN_956 LEU_957 ASP_958 SER_959 ARG_960 SER_961 PHE_962 LYS_963
GLN_964 PHE_965 PHE_966 GLN_967 LYS_968 PHE_969 ILE_970 GLN_971
ALA_972 LEU_973 LYS_974 SER_975
----------------
>2BYT:D --> 1OBH:A
GLY_154 LEU_155 VAL_156 ASN_157 TRP_158 CYS_159 PRO_160 LYS_161
CYS_162 GLN_163 THR_164 VAL_165 LEU_166 ALA_167 ASN_168 GLU_169
GLN_170 VAL_171 VAL_172 GLU_173 GLY_174 ARG_175 CYS_176 TRP_177
ARG_178 HIS_179 GLU_180 ASP_181 THR_182 PRO_183 VAL_184 GLU_185
LYS_186 ARG_187 GLU_188 LEU_189 GLU_440 ALA_441 CYS_442 GLY_443
ARG_466 PRO_467 LYS_468 GLY_469 LYS_470 LYS_486 CYS_487 GLY_488
GLY_489 PRO_490 ALA_491 ASP_815 VAL_816 VAL_817 GLU_818 VAL_819
ALA_820 VAL_821 GLN_822 VAL_823 ASN_824 GLY_825 ARG_826 VAL_827
ARG_828 GLY_829 THR_830 ILE_831 HIS_832 ILE_833 PRO_834 LYS_835
ASP_836 ALA_837 PRO_838 LEU_839 GLU_840 VAL_841 ALA_842 ARG_843
ALA_844 GLU_845 ALA_846 LEU_847 LYS_848 VAL_849 ARG_850 ASN_851
VAL_852 ARG_853 ALA_854 HIS_855 LEU_856 GLU_857 GLY_858 LYS_859
GLU_860 VAL_861 VAL_862 LYS_863 GLU_864 ILE_865 TYR_866 VAL_867
PRO_868 GLY_869 LYS_870 ILE_871 LEU_872 ASN_873 LEU_874 VAL_875
VAL_876
----------------
>2F8S:A --> 1YVU:A
GLU_262 ASN_263 LEU_264 GLU_265 ASP_266 GLU_267 TYR_362 LYS_363
LYS_364 LEU_365 GLU_366 GLU_426 TYR_427 PRO_428 LYS_429 VAL_430
ASP_431 PRO_432 TYR_433 LYS_434 SER_435
----------------
>3CIY:A --> 3CIG:A
ASN_550 PRO_551 GLY_552 GLY_553 PRO_554
----------------
>3UZS:A --> 1YM7:D
GLU_490 GLU_491 ASP_492 LYS_543 ASN_544 LYS_545 GLN_546 LEU_547
GLY_548 HIS_549 GLU_550 GLU_551 PRO_571 PHE_572 LEU_573 LEU_657
```

```
VAL_658 GLN_659 ARG_660 VAL_661 PRO_662 LYS_663 MET_664 LYS_665
ASN_666 LYS_667 PRO_668 ARG_669 ALA_670 PRO_671
----------------
>1F7U:A --> 1BS2:A
ALA_2 SER_3 THR_4
----------------
>4A36:B --> 4A2P:A
THR_243 LYS_244 LYS_245 ASN_331 PHE_332 SER_333 ASN_334 VAL_335
ALA_401 SER_402 GLN_403 LEU_662 MET_663 GLY_664 ARG_665 GLY_666
MET_674 THR_675 LEU_676 PRO_677 SER_678 GLN_679 LYS_680 GLY_681
VAL_682 LEU_683 ASP_684 ALA_685 PHE_686 LYS_687 THR_688 SER_689
LYS_690 ASP_691 VAL_700 ALA_701 ASP_702 GLU_703 GLY_704 ILE_705
ASP_706 ILE_707 VAL_708 GLN_709 CYS_710 ARG_729 GLY_730 ARG_731
GLY_732 ARG_733 ALA_734 ALA_735 GLY_736
----------------
>1YVP:A --> 1YVR:A
LYS_136 GLU_137 GLY_138 MET_139 LYS_140 CYS_141 GLY_142 MET_143
----------------
>1H4S:A --> 1HC7:A
ARG_78 LYS_79 GLU_80 ALA_81 GLU_82 HIS_83 VAL_84 GLU_85 GLY_86
----------------
>4YYE:B --> 4EO4:B
THR_119 ASP_120 GLU_121 GLU_122
----------------
>3OVS:B --> 1UET:A
SER_89 TYR_90 GLU_91 ILE_92 ARG_93 TYR_94 ALA_95
----------------
>5HC9:A --> 3H39:A
GLU_106 TYR_107 TYR_108 GLU_109 SER_110 PRO_111 ALA_112 LYS_113
LEU_114 PRO_115
----------------
>1QZW:A --> 3KL4:A
GLU_308 GLU_309 TYR_310 ASP_311 LYS_312 ILE_313 GLN_314 LYS_315
LYS_316 MET_317 GLU_318 ASP_319 VAL_320 MET_321 GLU_322 GLY_323
LYS_324 GLY_325 LYS_326
----------------
>1H3E:A --> 1H3F:A
THR_80 GLY_81 MET_82 ILE_83 GLY_84 ASP_85 PRO_86 SER_87 GLY_88 ARG_89
SER_90 LYS_91 THR_92 ARG_93 PRO_94 PRO_95 LEU_96 THR_97 LEU_98 GLU_99
GLU_100 GLY_348 GLY_349 ILE_350 PRO_351
----------------
>1YTU:A --> 1W9H:A
```

```
MSE_2 GLU_3 TYR_4 LYS_5 ILE_6 VAL_7 GLU_8 ASN_9 GLY_10 LEU_144 SER_145
ASN_146 ARG_147 ASN_148
----------------
>1HVU:H --> 2VG7:B
LYS_66 ASP_67 SER_68 THR_69 LEU_283 ARG_284 GLY_285 ARG_356 MET_357
ARG_358 GLY_359 ALA_360
----------------
>3KFU:A --> 1N9W:A
VAL_94 GLU_95 ILE_96 PRO_97 LYS_98 GLU_99 GLU_100 TRP_101 ARG_102
ALA_103 ASN_104 PRO_105 ASP_106 THR_107 LEU_108 LEU_109 GLU_110
----------------
>3V11:A --> 4M0L:B
SER_35 LYS_36 HIS_37 SER_38 GLU_39 GLU_40 LEU_41 LYS_42 ARG_43 GLY_44
PRO_226 GLY_227 THR_228 GLN_229 PHE_230 ASN_231 GLU_232 LEU_233
----------------
>1Q2R:A --> 4Q4R:A
SER_126 HIS_127 LEU_128 ASP_129 GLY_130 SER_131 ARG_132 ARG_384
ASN_385 SER_386
----------------
>3NMU:B --> 2NNW:A
LYS_297 ALA_298 LEU_299 PHE_300 ARG_301 HIS_302 LEU_303 ARG_304
THR_305 GLY_306 ALA_307 LYS_308 PRO_309
----------------
>4RWP:A --> 1PX5:A
THR_48 ALA_49 ASP_50 GLY_64 LYS_65 GLY_66 THR_67 THR_68 LEU_69 ARG_70
GLY_71 ARG_124 ARG_125 GLU_126 ASN_127 PRO_128 ARG_129
----------------
>1EIY:A --> 1B70:A
LEU_6 ALA_7 ALA_8 ILE_9 GLN_10 ASN_11 ALA_12 ARG_13 ASP_14 LEU_15
GLU_16 GLU_17 LEU_18 LYS_19 ALA_20 LEU_21 LYS_22 ALA_23 ARG_24 TYR_25
LEU_26 GLY_27 LYS_28 LYS_29 GLY_30 LEU_31 LEU_32 THR_33 GLN_34 GLU_35
MET_36 LYS_37 GLY_38 LEU_39 SER_40 ALA_41 LEU_42 PRO_43 LEU_44 GLU_45
GLU_46 ARG_47 ARG_48 LYS_49 ARG_50 GLY_51 GLN_52 GLU_53 LEU_54 ASN_55
ALA_56 ILE_57 LYS_58 ALA_59 ALA_60 LEU_61 GLU_62 ALA_63 ALA_64 LEU_65
GLU_66 ALA_67 ARG_68 GLU_69 LYS_70 ALA_71 LEU_72 GLU_73 GLU_74 ALA_75
ALA_76 LEU_77 LYS_78 GLU_79 ALA_80 LEU_81 GLU_82 ARG_83 GLU_84 ARG_85
----------------
>2ZZM:A --> 2YX1:B
LYS_69 ILE_70 ILE_71 LYS_72 LYS_73 PRO_74 SER_75 MLZ_81 MLZ_82 LYS_83
TYR_84 ARG_85 LYS_86 GLU_87 ILE_88 ASP_89 GLU_90 SER_138 GLU_139
VAL_140 LYS_141 GLY_142 GLU_143 PHE_144 ARG_145
----------------
>3X1L:B --> 4H4K:A
```

```
LEU_13 PHE_14 ARG_15 GLU_16 SER_17 ARG_18 PRO_19 PHE_20 ASP_21 ALA_22
GLY_23 SER_24 GLU_25 SER_26 VAL_27 ALA_28 ARG_29 SER_30 ILE_31 LYS_49
GLY_50 LEU_51 LYS_52 ASN_53 CYS_54 VAL_55 GLY_56 VAL_57 GLY_58 GLU_59
GLU_142 LYS_143 GLU_144 LYS_145 ARG_146 ILE_147 GLY_148 ILE_149
LYS_150 LEU_151 SER_152 ARG_153 GLU_154 LYS_155 LYS_156 VAL_157
VAL_158 GLU_159 GLU_160 GLY_161 MET_162 LEU_163 TYR_164 THR_165
VAL_166 GLU_167 PHE_168
----------------
>2GJW:A --> 1R0V:A
ILE_6 GLY_7 GLY_8 ASP_9 PHE_10 ALA_11 VAL_12 VAL_13 LYS_14 ALA_15
LYS_16 LYS_17 SER_18 LEU_19 GLU_20 ARG_21 ARG_22 GLY_23 PHE_24 GLY_25
VAL_26 LYS_27 ARG_28 GLY_29 ASP_30 LYS_31 ILE_32 TYR_33 LEU_34 HIS_35
PRO_36 LEU_37 GLU_38 VAL_39 VAL_40 TYR_41 LEU_42 GLN_43 ILE_44 LYS_45
GLY_46 ILE_47 GLU_48 SER_49 PHE_50 GLY_51 GLU_52 LEU_53 GLU_54 ASP_55
VAL_56 LEU_57 SER_58 TRP_59 ALA_60 GLU_61 SER_62 ARG_63 MET_64 GLU_65
----------------
>1J1U:A --> 1U7D:B
ARG_132 SER_133 MET_134 GLU_135 LEU_136 ILE_137 ALA_138 ARG_139
GLU_140 ASP_141 GLU_142 ASN_143 GLN_173 ARG_174 LYS_175
----------------
>4C9D:A --> 4C98:A
GLY_58 PRO_59 GLU_60 GLY_61 ALA_62 THR_63 LEU_64 LYS_65 LEU_88 GLU_89
ALA_90 ALA_91 PRO_92 ASP_93 GLY_94 LEU_95 PRO_96 LEU_97 GLY_98 ASP_99
PRO_149 GLY_150 GLY_151 ARG_152
----------------
>3RW6:A --> 3RW7:D
ASN_118 TRP_119 PHE_120 LYS_121 ILE_122 THR_123 ILE_124 PRO_125
TYR_126 GLY_127 ARG_128 LYS_129 TYR_130 ASP_131 LYS_132 ALA_133
TRP_134 LEU_135 LEU_136 SER_137 MET_138 ILE_139 GLN_140 SER_141
LYS_142 CYS_143 SER_144 VAL_145 PRO_146 PHE_147 THR_148 PRO_149
ILE_150 GLU_151 PHE_152 HIS_153 TYR_154 GLU_155 ASN_156 THR_157
ARG_158 ALA_159 GLN_160 PHE_161 PHE_162 VAL_163 GLU_164 ASP_165
ALA_166 SER_167 THR_168 ALA_169 SER_170 ALA_171 LEU_172 LYS_173
ALA_174 VAL_175 ASN_176 TYR_177 LYS_178 ILE_179 LEU_180 ASP_181
ARG_182 GLU_183 ASN_184 ARG_185 ARG_186 ILE_187 SER_188 ILE_189
ILE_190 ILE_191 ASN_192 SER_193 SER_194 ALA_195 PRO_196 PRO_197
HIS_198 THR_199 ILE_200 LEU_201 ASN_202 GLU_203
----------------
>4YVI:A --> 1UAJ:A
ASP_169 SER_170 PHE_171 ALA_172 ASP_173
----------------
>2XBM:A --> 4R8S:A
GLY_3 SER_4 GLN_5 GLY_6
```

```
----------------
>4LGT:D --> 4LAB:A
MET_1 SER_2 GLU_3 LYS_4 LEU_5 GLN_6 LYS_7 VAL_8 LEU_9 ALA_10 ARG_11
ALA_12 GLY_13 HIS_14 GLY_15 SER_16 ARG_17 ARG_18 GLU_19 ILE_20 GLU_21
SER_22 ILE_23 ILE_24 GLU_25 ALA_26 GLY_27 ARG_28 VAL_29 SER_30 VAL_31
ASP_32 GLY_33 LYS_34 ILE_35 ALA_36 LYS_37 LEU_38 GLY_39 ASP_40 ARG_41
VAL_42 GLU_43 VAL_44 THR_45 PRO_46 GLY_47 LEU_48 LYS_49 ILE_50 ARG_51
ILE_52 ASP_53 GLY_54 HIS_55 LEU_56 ILE_57 SER_58 VAL_59 ARG_60 GLU_61
----------------
>3FTF:A --> 3FTC:A
MET_1 VAL_2 ARG_3 LEU_4 LYS_5 LYS_6 SER_7 PHE_8 GLY_9 GLN_10 HIS_11
----------------
>3U56:A --> 3TG8:A
PRO_1 LYS_2 HIS_3 GLY_4 LYS_5 ARG_6 TYR_7 ARG_8 ALA_9 LEU_10 LEU_11
GLU_12 LYS_13
----------------
>2R8S:H --> 4XH2:H
LYS_222 SER_223 CYS_224
----------------
>4P3E:C --> 4P3F:A
ASN_138 THR_139 GLU_140 PRO_141
----------------
>4YHW:A --> 4YHU:C
GLU_407 LEU_408 HIS_409 THR_410 ASN_411 THR_412 GLY_413 ASP_414
ILE_415 LYS_416 MSE_417 ASP_418 MSE_419 HIS_420 ASN_421
----------------
>4OX9:Y --> 3P2K:A
SER_142 ASP_143 SER_144 TYR_145 GLU_146 GLU_147 ALA_148 GLU_149
ILE_150 LYS_151 LYS_152 ARG_153 GLY_154 LEU_155 PRO_156 LEU_157
LEU_158 SER_159 SER_217 LYS_218 HIS_219
----------------
>4ADV:J --> 3W1Y:C
SER_54 PRO_55 HIS_56 VAL_57 ASN_58 LYS_59 ASP_60 ALA_61 ARG_62
---------------
>3EQT:A --> 2W4R:D
PRO_594 VAL_595 VAL_596 ILE_597 ASN_598 LYS_599 VAL_600
----------------
>1ZBI:A --> 1ZBF:A
GLY_194 ARG_195 LYS_196
----------------
>4ERD:A --> 4EYT:A
GLU_520 PHE_521 ARG_522 LYS_523 PHE_524 PHE_525 PHE_526 MSE_527
LYS_528 LYS_529 GLN_530 GLN_531 LYS_532
```

```
----------------
>2QUX:A --> 2QUD:B
ASP_66 SER_67 GLY_68
----------------
>2CZJ:A --> 1WJX:A
TYR_63 GLU_64 LYS_65 GLY_66 SER_67 TYR_68 ALA_69 ASN_70
----------------
>4V2S:A --> 3VU3:E
PRO_67 VAL_68 SER_69 HIS_70 HIS_71 SER_72 ASN_73 ASN_74
----------------
>3WBM:A --> 2BKY:B
THR_5 PRO_6 THR_7 PRO_8
----------------
>1URN:A --> 1NU4:A
ALA_2 VAL_3 PRO_4 GLU_5 THR_6
----------------
>3MOJ:B --> 2G0C:A
LYS_414 LYS_415 ILE_416 ARG_417 LYS_468 GLY_469 LYS_470
----------------
```

Supplementary Figures

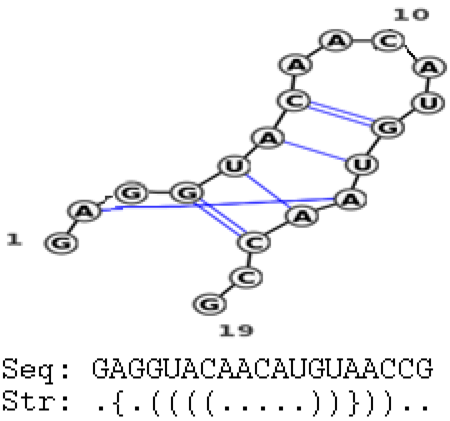

Figure S1. Secondary structure of RNA represented by dot bracket notation. The dot (".") represents an unpaired nucleotide, ("(" and ")") represents paired nucleotide and ("{" and "}") represent pseudoknot nucleotide.

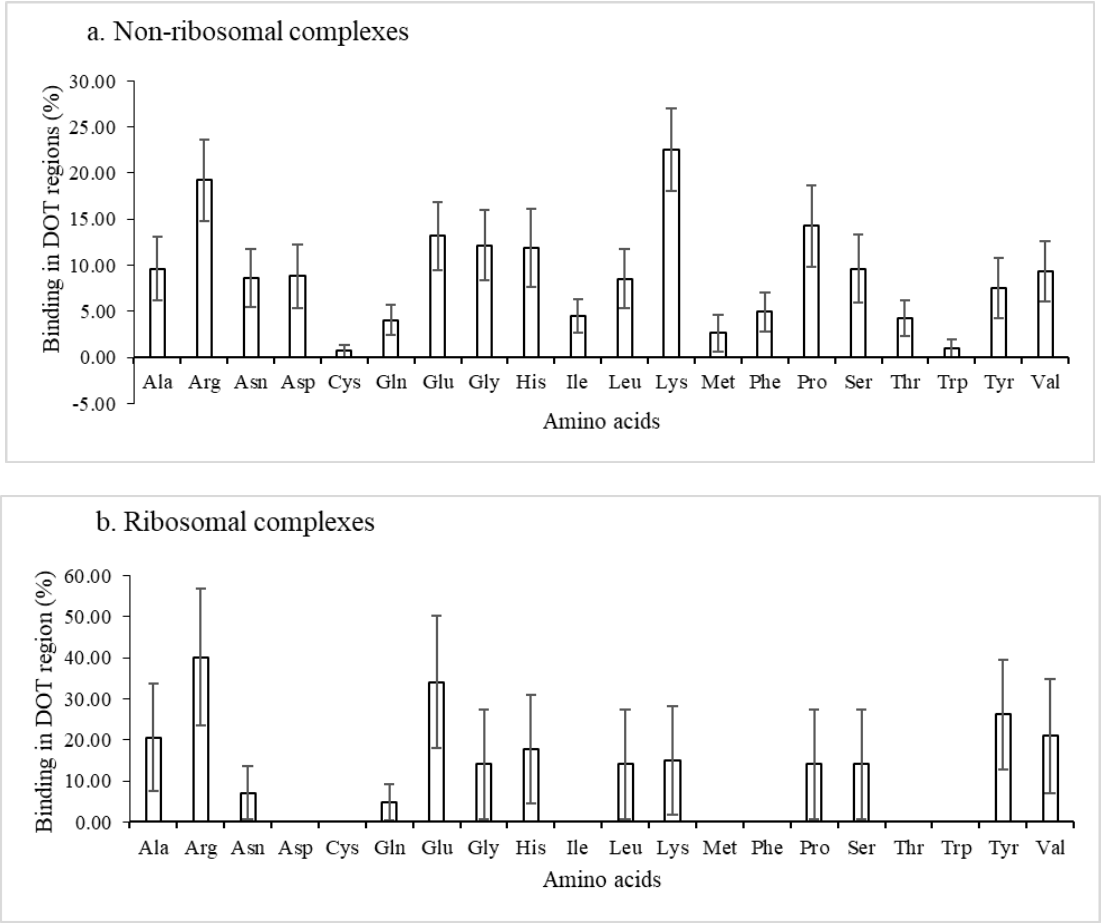

Figure S2. Frequency of binding of amino acids in DOT region for (**a**) non-ribosomal (NR6) and (**b**) ribosomal (RB6) protein-RNA complexes.

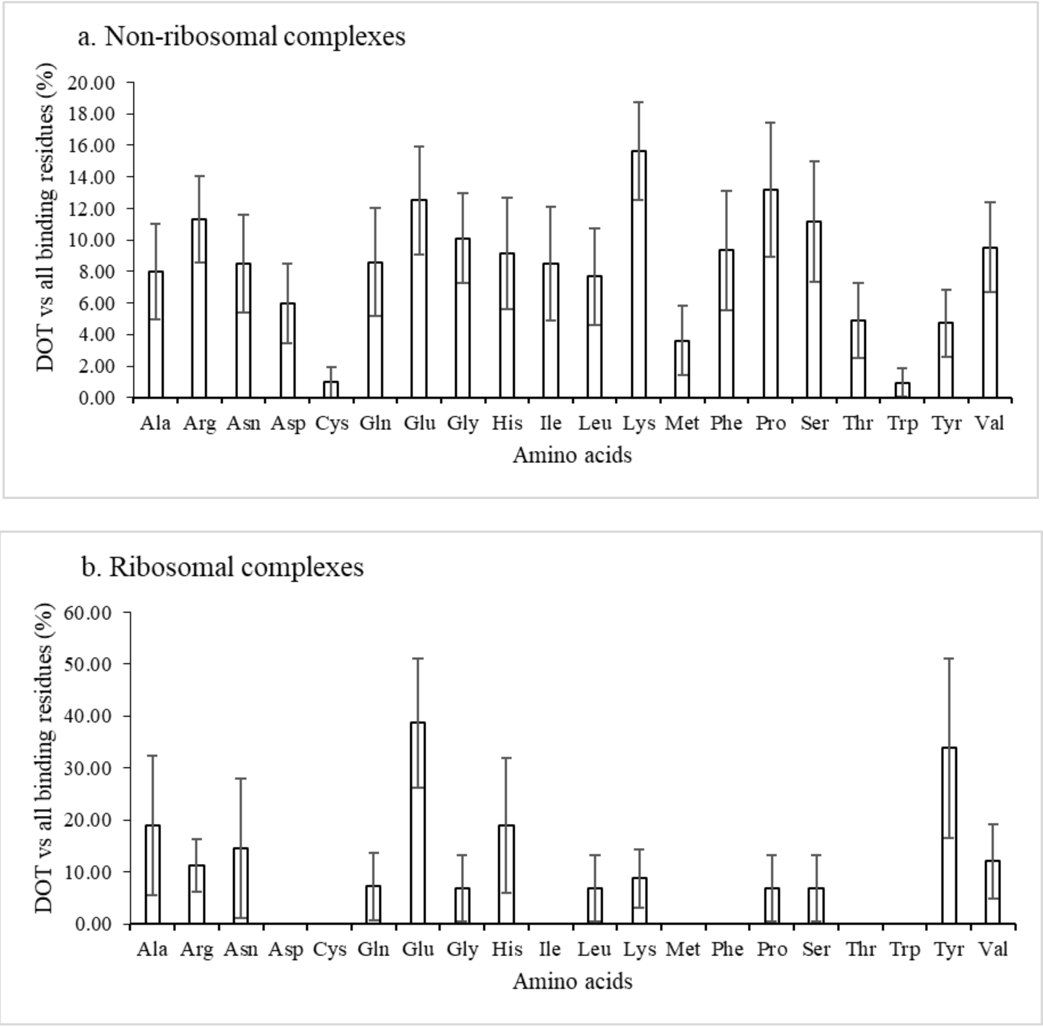

Figure S3. Frequency of binding in DOT regions than other binding residues in (**a**) non-ribosomal (NR6) and (**b**) ribosomal (RB6) protein-RNA complexes.

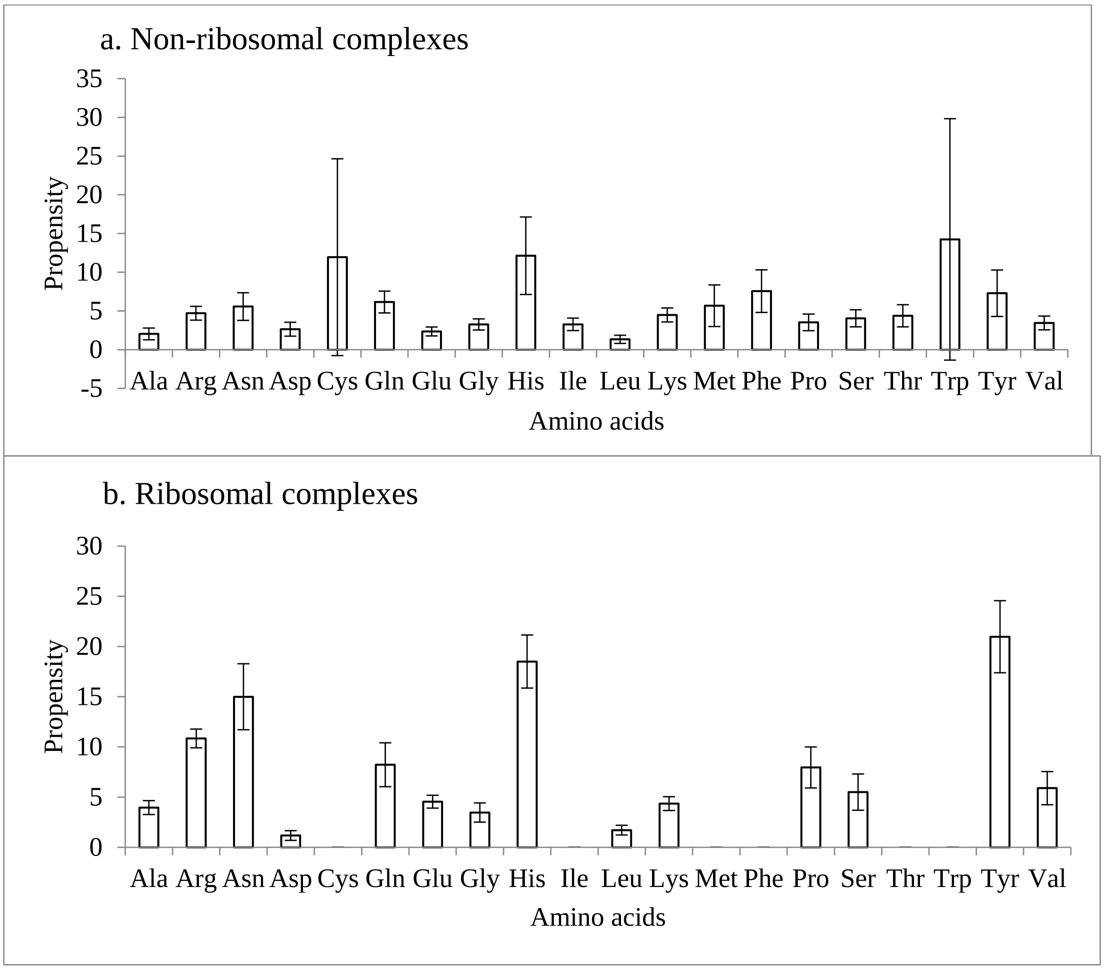

Figure S4. Propensity of binding in DOT region for (**a**). non-ribosomal and (**b**). ribosomal complexes using 6 Å distance cut-off for binding.

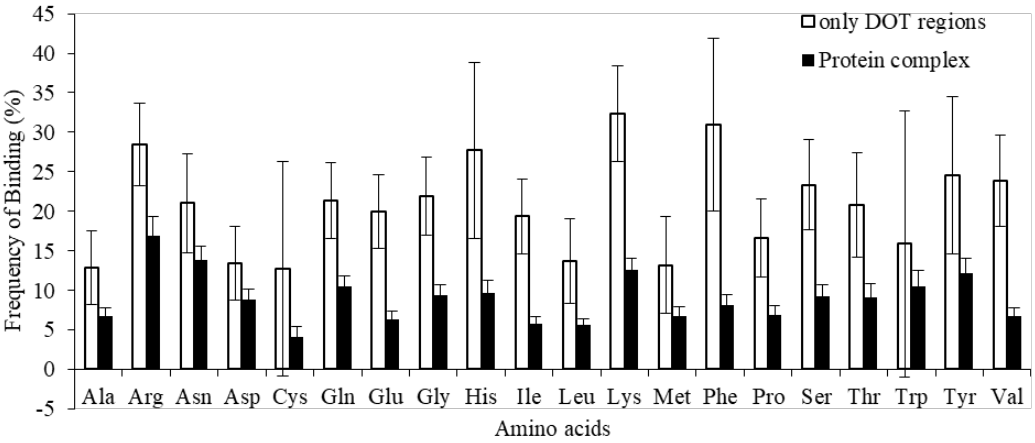

Figure S5. Comparison of binding frequency in DOT region and in complete protein using 6 Å distance cut-off for contact in NR6 dataset.

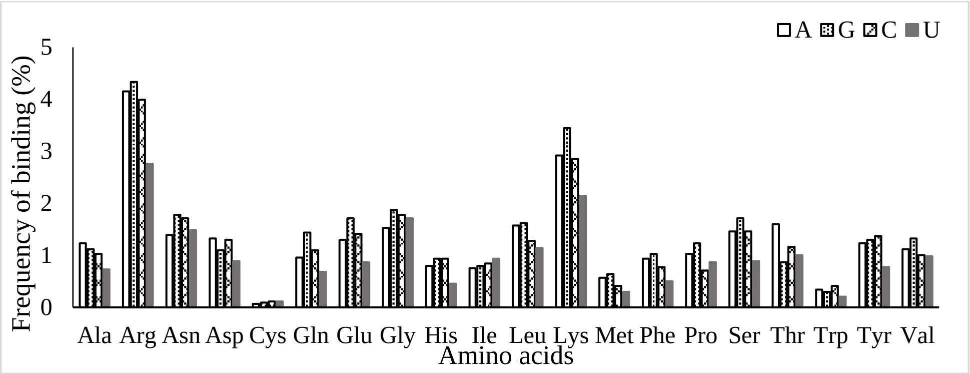

Figure S6. Frequency of binding of amino acids with nucleotides in non-ribosomal protein-RNA complexes using 6 Å distance for contact.

Supplementary Tables

Table S1. Secondary structure of all DOT residues and residues in contact with RNA in DOT region in non-ribosomal protein at 6 Å.

Secondary Structure	Number of Residues in Binding DOT Regions (N_{idt})	Number of Residues in DOT Region (N_d)	Relative Binding Residues in DOT Regions
Helix	58 (21.97)	288 (24.51)	20.14
Sheet	49 (18.56)	145 (12.34)	33.80
Others (Coil, turn, bend)	157 (59.47)	742 (63.15)	21.16

Note: Percentage is mentioned in parenthesis. Relative binding in DOT regions are calculated by $N_{idt}/N_d \times 100$.

Table S2. Number of nucleotides in contact with DOT regions and complete protein and their percentages in non-ribosomal proteins, at 6 Å cutoff.

Nucleotides	Number of Nucleotides in Contact with DOT Regions (N_{idt})	Number of Nucleotides in Contact with Any Residue of Proteins (N_{prot})	Relative Contacts in DOT Regions (%)
A	49 (22.90)	217 (25.35)	22.58
C	59 (27.57)	219 (25.58)	26.94
G	67 (31.31)	252 (29.44)	26.59
U	39 (18.22)	168 (19.63)	23.21

Note: Percentage is mentioned in parenthesis. Relative contact in DOT regions are calculated by $N_{idt}/N_{prot} \times 100$.

Table S3. Secondary structure of RNA interacting with DOT region in NR6 dataset.

**Nucleotide**	**Secondary Structure**	**Number of Nucleotides in Contact with DOT Regions (N_{idt})**	**Number of Nucleotides in Contact with Any Residue of Proteins (N_{prot})**	**Relative Contact in DOT Regions (%)**
A	Unpaired	40 (18.78)	168 (19.67)	23.81
A	Basepaired	9 (4.23)	43 (5.04)	20.93
A	Pseudoknot	0 (0)	0 (0)	0
C	Unpaired	23 (10.80)	102 (11.94)	22.55
C	Basepaired	35 (16.43)	113 (13.23)	30.97
C	Pseudoknot	1 (0.47)	6 (0.70)	16.67
G	Unpaired	28 (13.15)	125 (14.64)	22.40
G	Basepaired	37 (17.37)	126 (14.75)	29.37
G	Pseudoknot	1 (0.47)	4 (0.47)	25.00
U	Unpaired	27 (12.68)	115 (13.47)	23.48
U	Basepaired	12 (5.63)	51 (5.97)	23.53
U	Pseudoknot	0 (0)	1 (0.12)	0
All	Unpaired	118 (55.40)	510 (59.72)	23.14
All	Basepaired	93 (43.66)	333 (38.99)	27.93
All	Pseudoknot	2 (0.94)	11 (1.29)	18.18

Note: Percentage is mentioned in parenthesis. Relative contact in DOT regions are calculated by $N_{idt}/N_{prot} \times 100$.

© 2018 by the authors. Submitted for possible open access publication under the terms and conditions of the Creative Commons Attribution (CC BY) license (http://creativecommons.org/licenses/by/4.0/).
